# Children with hearing impairment in Malawi, a cohort study

**DOI:** 10.2471/BLT.18.226241

**Published:** 2019-05-28

**Authors:** Wakisa Mulwafu, Myroslava Tataryn, Sarah Polack, Asgaut Viste, Frederik Kragerud Goplen, Hannah Kuper

**Affiliations:** a Department of Surgery, College of Medicine, Private Bag 360, Blantyre, Malawi.; b International Centre for Evidence in Disability, London School of Hygiene & Tropical Medicine, London, England.; c Department of Clinical Medicine, Haukeland University Hospital, Bergen, Norway.

## Abstract

**Objective:**

To assess the outcomes of children diagnosed with hearing impairment 3 years earlier in terms of referral uptake, treatment received and satisfaction with this treatment, and social participation.

**Methods:**

We conducted a population-based longitudinal analysis of children with a hearing impairment in two rural districts of Malawi. Key informants within the community identified the cohort in 2013 (baseline). Informants clinically screened children at baseline, and by questionnaires at baseline and follow-up in 2016. We investigated associations between sociodemographic characteristics and outcomes by multivariate logistic regression.

**Results:**

We diagnosed 752 children in 2013 as having a hearing impairment and traced 307 (40.8%) children of these for follow-up in 2016. Referral uptake was low (102/184; 55.4%), more likely among older children (odds ratio, OR: 3.5; 95% confidence interval, CI: 1.2–10.2) and less likely for those with an illiterate caregiver (OR: 0.5; 95% CI: 0.2–0.9). Few of the children who attended hospital received any treatment (33/102; 32.4%) and 63.6% (21/33) of caregivers reported satisfaction with treatment. Difficulty making friends and communicating needs was reported for 10.0% (30/299) and 35.6% (107/301) of the children, respectively. Lack of school enrolment was observed for 29.5% (72/244) of children, and was more likely for older children (OR: 28.6; 95% CI: 10.3–79.6), girls (OR: 2.4; 95% CI: 1.2–4.8) and those with an illiterate caregiver (OR: 2.1; 95% CI: 1.0–4.1).

**Conclusion:**

More widespread and holistic services are required to improve the outcomes of children with a hearing impairment in Malawi.

## Résumé

**Objectif:**

Évaluer la situation des enfants chez lesquels une déficience auditive a été diagnostiquée 3 années plus tôt, en matière de consultation d'un spécialiste, de traitement reçu et de satisfaction quant à ce traitement, ainsi que de participation sociale.

**Méthodes:**

Nous avons réalisé une analyse longitudinale dans la population des enfants présentant une déficience auditive dans deux districts ruraux du Malawi. Des informateurs clés de la communauté ont défini la cohorte en 2013 (point de référence). Ils ont pratiqué un examen clinique chez les enfants à cette date, et ont eu recours à des questionnaires à cette époque et lors du suivi en 2016. Nous avons étudié les associations entre certaines caractéristiques sociodémographiques et la situation des enfants à l'aide d'une régression logistique multivariée.

**Résultats:**

Nous avons diagnostiqué une déficience auditive chez 752 enfants en 2013 et avons fait le suivi de 307 (40,8%) d'entre eux en 2016. Le taux de consultation d'un spécialiste était faible (102/184; 55,4%), plus fréquent chez les enfants les plus âgés (rapport des cotes, RC: 3,5; intervalle de confiance de 95%, IC: 1,2–10,2) et moins fréquent chez ceux à charge d'une personne analphabète (RC: 0,5; IC 95%: 0,2–0,9). Parmi les enfants qui se sont rendus à l'hôpital, peu ont reçu un traitement (33/102; 32,4%) et 63,6% (21/33) des accompagnateurs se sont dits satisfaits du traitement. Des difficultés à se faire des amis et à communiquer ses besoins ont été rapportées respectivement par 10,0% (30/299) et 35,6% (107/301) des enfants. L'absence de scolarisation a été observée pour 29,5% (72/244) des enfants, plus fréquemment chez les plus âgés (RC: 28,6; IC 95%: 10,3-79,6), les filles (RC: 2,4; IC 95%: 1,2-4,8) et les enfants à charge d'une personne analphabète (RC: 2,1; IC 95%: 1,0-4,1).

**Conclusion:**

Il est nécessaire de proposer des services plus complets et généralisés pour améliorer la situation des enfants présentant une déficience auditive au Malawi.

## Resumen

**Objetivo:**

Evaluar los resultados de los niños diagnosticados con hipoacusia hace tres años con respecto a la asimilación de la remisión, el tratamiento recibido y la satisfacción con este tratamiento, y la participación social.

**Métodos:**

Se realizó un análisis longitudinal basado en la población de niños con discapacidad auditiva en dos distritos rurales de Malawi. Los informantes clave dentro de la comunidad identificaron a la cohorte en 2013 (inicio del estudio). Los informantes examinaron clínicamente a los niños y aplicaron cuestionarios desde el inicio, y luego el seguimiento se realizó en 2016. Se investigaron las asociaciones entre las características sociodemográficas y los resultados mediante la regresión logística multivariada.

**Resultados:**

En 2013, diagnosticamos a 752 niños con discapacidad auditiva y en 2016 seguimos el rastro de 307 (40,8 %) de ellos para su seguimiento. La asimilación de la remisión fue baja (102/184; 55,4 %), más probable entre los niños mayores (razón de momios, OR: 3,5; intervalo de confianza del 95 %, IC: 1,2-10,2) y menos probable entre los que tenían un cuidador analfabeto (OR: 0,5; IC del 95 %: 0,2-0,9). Pocos de los niños que asistieron al hospital recibieron tratamiento (33/102; 32,4 %) y 63,6 % (21/33) de los cuidadores informaron satisfacción con el tratamiento. La dificultad para hacer amigos y comunicar las necesidades fue reportada para el 10.0 % (30/299) y 35.6 % (107/301) de los niños, respectivamente. La falta de escolarización se observó en el 29,5 % (72/244) de los niños, y fue más probable en los niños mayores (OR: 28,6; IC del 95 %: 10,3-79,6), las niñas (OR: 2,4; IC del 95 %: 1,2-4,8) y los que tenían un cuidador analfabeto (OR: 2,1; IC del 95 %: 1,0-4,1).

**Conclusión:**

Se necesitan servicios más amplios y holísticos para mejorar los resultados de los niños con discapacidad auditiva en Malawi.

## ملخص

**الغرض:**

تقييم للنتائج الخاصة بالأطفال الذين تم تشخيص إصابتهم بضعف السمع منذ 3 سنوات، فيما يتعلق بالحصول على فرصة للإحالة، وتلقى العلاج، والرضا عن هذا العلاج، والمشاركة الاجتماعية.

**الطريقة:**

قمنا بإجراء تحليلاً طولانياً على أساس السكان من الأطفال الذين يعانون من ضعف السمع في اثنين من المناطق الريفية في ملاوي. يمثل المبلغون الرئيسيون في المجتمع مجموعة الأتراب في عام 2013 (خط الأساس). قام المبلغون بفحص الأطفال سريرياً عند خط الأساس، ومن خلال الاستبيانات عند الأساس والمتابعة في عام 2016. وقمنا بفحص الارتباطات بين الخصائص الاجتماعية الديموغرافية ونتائج التحوف اللوجستي متعدد المتغيرات.

**النتائج:**

لقد قمنا بتشخيص 752 طفلًا في عام 2013 على أنهم يعانون من ضعف في السمع، وقمنا بتتبع 307 طفلاً (40.8%) منهم للمتابعة في عام 2016. كانت نسبة الحصول على فرصة الإحالة منخفضة (102/184؛ 55.4%)، وكانت أكثر احتمالية بين الأطفال الأكبر سناً (نسبة الاحتمالات: 3.5؛ فاصل الثقة 95%: 1.2 إلى 10.2)، واحتمالية أقل لهؤلاء الأطفال ممن يخضعون لرعاية شخص أُمي (نسبة الاحتمالات: 0.5؛ ‏بفاصل ثقة 95%: 0.2 إلى 0.9). عدد قليل من الأطفال الذين تلقوا الرعاية في المستشفى، وحصلوا على أي علاج (33/102؛ بنسبة 32.4%) وأعربت نسبة 63.6% (21/33) من مقدمي الرعاية عن رضاهم عن العلاج. تم الإبلاغ عن صعوبة في تكوين الصداقات، واحتياجات التواصل لنسبة 10.0% (30/299)، و35.6% (107/301) من الأطفال، على الترتيب. تمت ملاحظة نقص في الالتحاق بالتعليم لنسبة 29.5% (72/244) من الأطفال، وكانت أكثر احتمالية بالنسبة للأطفال الأكبر سناً (نسبة الاحتمالات: 28.6؛ ‏بفاصل ثقة 95%: 10.3 إلى 79.6)، والبنات (نسبة الاحتمالات: 2.4؛ ‏بفاصل ثقة 95%: 1.2 إلى 4.8)، وهؤلاء الأطفال ممن يخضعون لرعاية شخص أُمي (نسبة الاحتمالات: 2.1؛ ‏بفاصل ثقة 95%: 1.0 إلى 4.1).

**الاستنتاج:**

هناك حاجة إلى خدمات أكثر انتشاراً وشمولية لتحسين نتائج الأطفال المصابين بضعف السمع في ملاوي. ****

## 摘要

**目的:**

从转诊率、接受治疗情况、对治疗的满意程度及社会参与度等方面对 3 年前确诊为听力障碍儿童的治疗结果进行评估。

**方法:**

我们对马拉维两大农村地区中患有听力障碍的儿童进行了一项基于人口的纵向分析。我们确定了 2013 年社区内关键受访者群组（基准值）。受访者在 2016 年通过基准值调查问卷和随访的形式对儿童进行了临床筛查。我们采用多变项逻辑回归法对社会人口特征与结果之间的关系进行了研究。

**结果:**

我们在 2013 年诊断出 752 名儿童患有听力障碍，并在 2016 年对其中的 307 名儿童 (40.8%) 进行了随访。转诊率较低（102/184；55.4%），且多见于年龄较大的儿童（比值比，OR: 3.5；95% 置信区间，CI：1.2-10.2），而其看护人未受过教育的儿童转诊率更低（比值比，OR：0.5；95％ 置信区间，CI：0.2-0.9）。只有极少数在医院就诊的儿童接受了治疗（33/102；32.4%），其中 63.6% (21/33) 的看护人对治疗表示满意。报告表明，10.0% (30/299) 的儿童交友困难，35.6% (107/301) 的儿童有沟通障碍。据悉，儿童失学率达到 29.5% (72/244)，其中年龄较大的儿童（比值比，OR：28.6；95％ 置信区间，CI：10.3-79.6）、女孩（比值比，OR：2.4；95％ 置信区间，CI：1.2-4.8）以及其看护人未接受过教育的儿童（比值比，OR：2.1；95% 置信区间，CI：1.0-4.1）的失学率更高。

**结论:**

马拉维仍需要一系列更广泛的服务来改善患有听力障碍儿童的治疗结果。

## Резюме

**Цель:**

Оценка результатов вмешательства у детей, у которых за три года до этого были диагностированы нарушения слуха, применительно к использованию направлений к врачам-специалистам, полученному лечению и удовлетворенности его результатами, а также социальному участию.

**Методы:**

Авторы провели популяционное лонгитюдное исследование детей с нарушениями слуха в двух сельских районах Малави. Основные информанты в сообществе определили когорту в 2013 году (базовые данные). Информанты провели клинический скрининг детей в момент сбора базовых данных, а также анкетирование в момент сбора базовых данных и повторно в 2016 году. Авторы исследовали взаимосвязь между социодемографическими характеристиками и результатами вмешательства с использованием множественной логистической регрессии.

**Результаты:**

Диагноз нарушений слуха был поставлен 752 детям в 2013 году, из них 307 детей (40,8%) участвовали в последующем контроле в 2016 году. Использование направлений к специалистам было низким (102 из 184; 55,4%). Вероятность обращения к врачу была выше для детей старшего возраста (отношение шансов, ОШ: 3,5; 95%-й ДИ: 1,2–10,2) и ниже в том случае, если осуществляющее уход лицо было неграмотным (ОШ: 0,5; 95%-й ДИ: 0,2–0,9). Немногие из детей, посетивших больницу, прошли курс лечения (33 из 102; 32,4%), а 63,6% (21 из 33) лиц, осуществлявших уход за детьми, сообщили о том, что они довольны лечением. Сложность в приобретении друзей и проблемы в общении отмечались у 10,0% (30 из 299) и 35,6% (107 из 301) детей соответственно. Недостаточная посещаемость школы отмечалась у 29,5% (72 из 244 детей) и была выше у детей старшего возраста (ОШ: 28,6; 95%-й ДИ: 10,3–79,6), девочек (ОШ: 2,4; 95%-й ДИ: 1,2–4,8) и в случаях, когда осуществляющее уход лицо было неграмотным (ОШ: 2,1; 95%-й ДИ: 1,0–4,1).

**Вывод:**

Для улучшения результатов лечения детей с нарушениями слуха в Малави необходима система повсеместного и комплексного медицинского обслуживания.

## Introduction

Approximately 466 million people live with disabling hearing loss globally, including 34 million children, and most of these live in low- and middle-income countries.^1^ Unaddressed hearing loss has a negative impact on language development, school performance, employment opportunities, psychosocial well-being and aspects of family life, with an estimated annual global cost to society of 750 billion United States dollars.^2^ Hearing loss often goes unnoticed and unaddressed, and its impact has not been explored adequately in low- and middle-income countries.^3^^,^^4^

Early detection, treatment and rehabilitation are important to mitigate some of these negative effects and maximize functioning for affected individuals. In 2017, the World Health Organization (WHO) adopted a resolution on ear and hearing care that urges Member States to develop, implement and monitor screening programmes for early identification of ear diseases and hearing loss in high-risk populations, including infants and young children.^5^ Ultimately, these initiatives contribute to the attainment of sustainable development goals 3 (that is, ensure healthy lives and promote well-being for all at all ages) and 4 (that is, ensure inclusive and equitable quality education and promote lifelong learning opportunities for all). However, in many low- and middle-income countries there is a shortage of good-quality ear and hearing services,^6^ and even when services are available utilization remains low.^7^^,^^8^ At national and regional levels, data are currently lacking on the need for ear and hearing services that would help to advocate for, plan and implement these programmes.

Data on the prevalence and causes of hearing loss in sub-Saharan Africa are sparse.^9^ In Malawi, a low-income country in southern Africa, there are two ear, nose and throat surgeons and three audiologists to serve a population of approximately 17.6 million people.^10^ A single community-based study has reported that the prevalence of childhood hearing impairment is high in Malawi, with 32/279 (11.5%) of children aged 4–6 years having bilateral hearing loss of greater than 25 decibels (dB) hearing level.^11^ Information on the broader impacts of hearing loss, referral uptake and the outcomes of treatment is lacking. Timely and regular follow-up of children with hearing loss is important, but often difficult to achieve in low- and middle-income countries.^12^^,^^13^ To provide a more comprehensive assessment of the impact of ear and hearing disorders, treatment outcomes should focus not only on formal diagnostic assessments and treatment received, but also on holistic assessments of children, such as well-being and education inclusion.

Here we aimed to assess the outcome of children with ear and hearing disorders 3 years after initial diagnosis, in terms of referral uptake, treatment received and satisfaction with this treatment. We also aimed to assess the social participation of the affected children, specifically, their ability to make friends and communicate needs, and their enrolment at school.

## Methods

### Study design and setting

Our hearing impairment investigation was part of a larger population-based study to estimate the prevalence of hearing, visual, physical and intellectual impairment and epilepsy in children in Malawi by the key informant method.^14^ The key informant method is a two-stage process including identification of children with impairments by key informants, followed by assessment of these children by relevant medical professionals at one of the 33 screening camps set up within the study area for the period April–November 2013. 

We selected the two rural districts of Thyolo (Southern Region, 18 camps) and Ntcheu (Central Region, 15 camps) for our study to enable us to achieve the desired target population of 1 million people. These districts are located relatively close to Blantyre, the commercial capital of Malawi. Children were identified through this study could benefit from referral to the community-based rehabilitation facility in Ntcheu and the outreach and inpatient services provided in Thyolo by centralized centres in Blantyre (e.g. Queen Elizabeth Central Hospital), through links with the Christian Blind Mission and Malawi Council for the Handicapped.

We focused our longitudinal analysis on the population-based sample of children confirmed by audiologists at the screening camps as having a hearing impairment. Trained key informants interviewed parents or caregivers and completed questionnaires at baseline in 2013, and trained community health workers conducted the follow-up survey in 2016. Before the baseline survey, we conducted a comprehensive mapping of the available referral services through discussions with local stakeholders and service providers. This mapping was essential to ensure the availability of services needed to accommodate additional demand generated by the study.

### Key informants

We selected a total of 500 literate key informants (250 per district) from existing pools of volunteers who work alongside health surveillance assistants, a formal cadre of community health workers in Malawi, to cover all the communities within the two districts. All key informants were trusted members within the community, but without formal expertise related to ear health and hearing. 

We trained the volunteers in groups of approximately 25 key informants per session (holding 10 training workshops per district) at a 4–5-hour workshop that included disability sensitization, identification of key impairments (including hearing), methods for case finding and procedures for the screening camps. We delivered training using specially designed flipcharts and hand-out information sheets produced in the local language (Chichewa) that contained information and illustrations regarding the impairments to be identified (including hearing), and instructions on how to conduct case finding and complete the registration forms. We based these materials on those developed and validated by the International Centre for Evidence in Disability that were previously used in studies conducted in Bangladesh and Pakistan.^15^

In 2013, trained key informants identified children suspected of having a disability (including hearing impairment) by spreading the word through their daily activities and existing social and professional networks, and visiting the homes of such children. The key informants then referred such children to the screening camps for clinical investigation by a team of specialists, including ear, nose and throat practitioners and audiologists. 

### Screening camps

Using funding from the Christian Blind Mission, the College of Surgeons of East Central and Southern Africa Oxford Orthopaedic Link programme, Cure International UK, Fight for Sight and the Liliane Foundation, we set up screening camps at which children suspected of having an impairment could be assessed. Screening camps were usually open for a single 8–12-hour day, unless demand was sufficiently high for a second day. A single team of medical professionals, comprising up to three from each of the different specialities (orthopaedic clinical officers, ear, nose and throat clinical officers, audiologists, ophthalmic clinical officers, nurses, social workers and rehabilitation technicians), attended each camp in turn. This attendance was on a voluntary basis and additional to normal medical duties. To assist the team, we delivered a 1-day training course on the organization of the screening camps and clinical examination protocols. Field supervisors (a Malawi key informant method project coordinator and a researcher from the London School of Hygiene & Tropical Medicine) attended the screening camps to monitor the quality of data collection and ensure consistency.^14^

On presentation at a screening camp, caregivers were asked a series of six questions to determine the type of medical assessment required. Audiologists used the WHO ear and hearing disorders survey protocol^16^ in the assessment of children suspected of having a hearing impairment. Audiologists conducted otoacoustic emission tests for children aged 6 months–4 years, and hearing impairment was defined as failed otoacoustic emission screening in both ears. For children aged 5–18 years, we attempted to use pure tone audiometry with a KUDUwave 5000 audiometer (eMoyoDotnet (Pty) Ltd, Randburg, South Africa). Pure tone audiometry requires the active cooperation of the child being tested, however, and some children aged 5–18 years could not be tested in this way; we therefore performed otoacoustic emission screening on some children of age 5–18 years. We diagnosed a hearing impairment for a hearing level of greater than 25 dB in the better hearing ear averaged across the frequencies of 0.5, 1, 2 and 4 kHz. The categories of hearing impairment were defined as: mild, 26–40 dB hearing level; moderate, 41–60 dB hearing level; severe, 61–80 dB hearing level; and profound, over 81 dB hearing level.^17^

The audiologist referred all children with hearing loss or ear disease to ear, nose and throat services, while explaining to the parent or caregiver what the findings were, why a referral was appropriate, how the referral could be pursued and what would happen at the appointment.

### Baseline survey

Key informants completed an initial questionnaire by interviewing the parent or caregiver. The covariates were guided by evidence from published literature,^18^^–^^20^ and included sociodemographic variables such as age, sex, location, income group, school enrolment, whether the child had a speech impairment and the literacy of the parent or caregiver. Speech impairment was defined as a caregiver’s response of “no” to “does the child have speech or vocalization?” (children aged < 2 years) or “can the child say names of familiar objects or speak whole sentences?” (children aged ≥ 2 years). Any child whose speech was different from or poorer than other children of the same age as reported by the caregiver was also categorized as having a speech impairment.

### Follow-up survey

In 2016, we conducted a follow-up of all the children originally identified as having a hearing impairment in 2013. We requested the assistance of key informants in tracing relevant children in their respective villages, using baseline data including the child’s name, age, sex, village of residence, contact number if any, and names of next of kin and relevant key informants. Our research assistants and the key informants worked together with community health workers in each of the relevant villages.

We delivered a 1-day training course to community health workers in tracing the children and administering a questionnaire to parents or caregivers during a home visit. The questionnaire included referral status (whether a referral was made and uptake of referral), treatment received and satisfaction with this treatment, ear and hearing status at follow-up (self-reported; caregivers were asked the question “Does he/she have difficulties in hearing sounds, such as people’s voices or music?”), speech and language difficulties, and participation outcomes. Participation outcomes were linked to the framework of the International Classification of Functioning, Disability and Health for Children and Youth,^20^ and measured using ability to make friends (d750, forming relationships) and communicate needs (d310, communicating with-receiving spoken messages; d315, communicating with-receiving nonverbal messages), and whether they were enrolled at school (d820, school education). In this case, school included primary- (for children aged 6 years and older), secondary- and university-level education.

We maintained contact with community health workers by mobile telephone text, assisting where children could not be traced. We also assessed loss to follow-up, defined as those who could not be traced 3 years after initial identification. 

### Data management and analysis

We entered all baseline data into an Access database (Microsoft, Redmond, United States of America). We double-entered 722/7220 (10.0%) of the forms and compared these to verify the quality of the data entry. We entered follow-up data into an Excel spreadsheet (Microsoft). We undertook data cleaning and analyses using Stata version 15 (StataCorp LCC, College Station, USA). We investigated the associations between children achieving an outcome (referral uptake, well-being and inclusion, and school enrolment) in terms of sociodemographic characteristics, such as literacy of caregiver, income group and whether the child had a speech impairment. We calculated odds ratios (OR) and 95% confidence intervals (CI) for the associations using a multivariate logistic regression model with stepwise backward selection. To reduce the chance of missing variables that could be relevant, a liberal *P*-value of 0.20 or less was chosen for inclusion in the model; factors that did not contribute to the model (*P* > 0.20) were eliminated to calculate an adjusted OR (aOR).

### Ethics

We obtained ethical approval from the College of Medicine Research Ethics Committee, Malawi and the London School of Hygiene & Tropical Medicine, United Kingdom of Great Britain and Northern Ireland. All parents gave written consent for inclusion in the study. If caregivers were illiterate, then the information sheet was read to them and they gave consent by thumb print. Caregivers were informed that participation in the study was voluntary, that refusal to participate would not affect any medical care they would receive and that they could discontinue participation at any time.

## Results

**Fig. 1:**
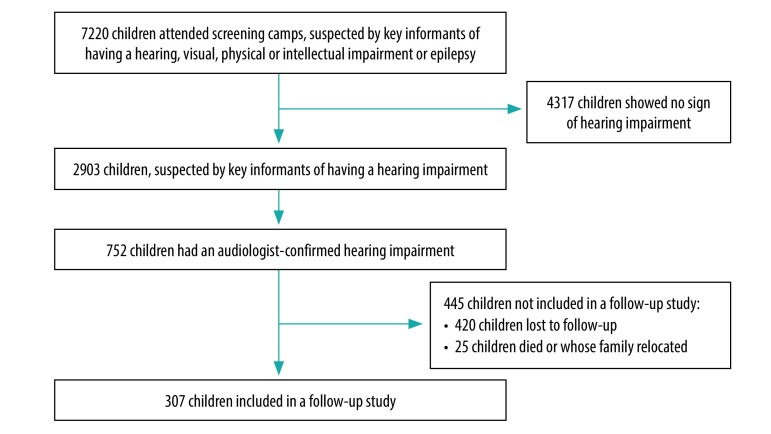
Numbers of children identified as having a hearing impairment by key informants in 2013 and traced to follow-up in 2016, Malawi

Of an estimated 15 000 children suspected by key informants as having either a hearing, visual, physical or intellectual impairment or epilepsy, 7220 (48%) attended one of the 33 screening camps. The key informants identified 2903 children as having a suspected hearing impairment, which was confirmed by audiologists in 752 children. Three years after baseline, we traced 307 (40.8%) of these children (Fig. 1). There was no significant difference between the groups included at baseline and at follow-up, except that children aged 15–18 years were slightly more likely to be included in the follow-up (OR: 1.7; 95% CI: 1.1–2.8), demonstrating that those followed up were relatively representative of the baseline group (Table 1). At baseline, 159 out of 310 (51.3%) of the children with hearing loss who underwent pure tone audiometry had moderate to profound hearing loss, and the remainder had mild hearing loss (Table 1).

**Table 1:** Sociodemographic characteristics of children identified with hearing impairment at baseline in 2013 and traced for follow-up in 2016, Malawi

Characteristic as recorded at baseline	No. at baseline	No. in follow-up (%)	OR (95% CI)
**Total**	**752**	**307 (40.8)**	**NA**
**Age at baseline, years**			
0–4	169	63 (37.3)	Reference
5–9	264	98 (37.1)	1.0 (0.7–1.5)
10–14	210	91 (43.3)	1.3 (0.9–2.0)
15–18	109	55 (50.5)	1.7 (1.1–2.8)
**Sex**			
Female	342	137 (40.1)	Reference
Male	410	170 (41.5)	1.1 (0.8–1.4)
**District**			
Thyolo	444	176 (39.6)	Reference
Ntcheu	308	131 (42.5)	1.1 (0.8–1.5)
**Degree of hearing loss**			
Mild	151	66 (43.7)	Reference
Moderate	138	63 (45.7)	1.1 (0.7–1.7)
Severe to profound	21	13 (61.9)	2.1 (0.8–5.4)
Bilateral otoacoustic emission failure	442	165 (37.3)	
**Causes of hearing loss**			
Ear infection			
Yes	510	205 (40.2)	0.9 (0.7–1.3)
No	242	102 (42.1)	Reference
Impacted wax			
Yes	250	97 (38.8)	0.9 (0.7–1.2)
No	502	210 (41.8)	Reference
Sensorineural			
Yes	188	78 (41.5)	1.0 (0.7–1.5)
No	564	229 (40.6)	Reference
**Speech impairment**			
Yes	179	73 (40.8)	1.0 (0.7–1.4)
No	573	234 (40.8)	Reference

CI: confidence interval; NA: not applicable; OR: odds ratio.

Of the 307 children included at follow-up, 184 (59.9%) were reported by the caregiver as having been referred to the district hospital at the original screening camp. Approximately half (102, 55.4%) of those referred to the district hospital reported that they had attended the district hospital for their referral. After eliminating non-significant variables (sex, whether enrolled in school, whether a speech impairment, income and degree of hearing loss), referral uptake was lower among children living in Ntcheu district (aOR: 0.4; 95% CI: 0.2–0.8) and among those with caregivers who were illiterate (aOR: 0.5; 95% CI: 0.2–0.9). Uptake was higher in the older age groups of 15–18 years (aOR: 3.5; 95% CI: 1.2–10.2; Table 2). Regarding intervention or treatment given, only nine patients received hearing aids, 15 underwent surgery and nine received special needs education. The caregivers of 63.6% (21/33) of the children who received any treatment reported that they were satisfied.

**Table 2:** Sociodemographic characteristics of children with hearing impairment whose caregiver reported referral uptake, Malawi, 2013 and 2016

Characteristic as recorded at baseline	No. referred to district hospital (*n* = 184)	No. (%) who attended hospital (*n* = 102)	cOR (95% CI)	aOR (95% CI)^a^
**Age, years**				
0–4	38	17 (44.7)	Reference	Reference
5–9	55	29 (52.7)	1.4 (0.6–3.2)	1.1 (0.5–2.8)
10–14	56	31 (55.4)	1.5 (0.7–3.5)	1.7 (0.7–4.2)
15–18	35	25 (71.4)	3.1 (1.2–8.3)	3.5 (1.2–10.2)
**Sex**				
Female	80	39 (48.8)	0.6 (0.3–1.1)	NA
Male	104	63 (60.6)	Reference	NA
**District**				
Ntcheu	93	42 (45.2)	0.4 (0.2–0.8)	0.4 (0.2–0.8)
Thyolo	91	60 (65.9)	Reference	Reference
**School enrolment**				
Yes	112	61 (54.5)	0.8 (0.3–1.9)	NA
No	22	13 (59.1)	Reference	NA
Not of school age^b^	50	28 (56.0)	NA	NA
**Speech impairment**				
Yes	46	30 (65.2)	1.7 (0.9–3.5)	NA
No	138	72 (52.2)	Reference	NA
**Illiterate caregiver**				
Yes	61	25 (41.0)	0.4 (0.2–0.8)	0.5 (0.2–0.9)
No	111	70 (63.1)	Reference	Reference
Not recorded	12	7 (58.3)	NA	NA
**Income group, MWK**				
≤ 12 000	165	89 (53.9)	0.4 (0.1–1.4)	NA
> 12 000	15	11 (73.3)	Reference	NA
Not recorded	4	2 (50.0)	NA	NA
**Degree of hearing loss^c^**				
Mild	41	24 (58.5)	Reference	NA
Moderate	45	26 (57.8)	1.0 (0.4–2.3)	NA
Severe to profound	6	4 (66.7)	1.4 (0.2–8.8)	NA
Bilateral otoacoustic emission failure	92	48 (52.2)	NA	NA

aOR: adjusted odds ratio; CI: confidence interval; cOR: crude odds ratio; MWK: Malawian Kwacha; NA: not applicable.

^a^ After eliminating non-significant variables (sex, whether enrolled in school, whether a speech impairment, income and degree of hearing loss).

^b^ School age is defined as 6 years or older.

^c^ Categories of hearing loss are defined as: mild, 26–40 dB hearing level; moderate, 41–60 dB hearing level; severe to profound, 61 dB hearing level and greater.

Note: Children were referred in 2013 and followed-up in 2016.

Of the 307 children included at follow-up, whether the child had difficulty making friends or communicating needs was not recorded for eight and six children, respectively. Children experiencing difficulties making friends was reported by 10.0% (30/299) of the caregivers. After eliminating non-significant variables (age, sex, district, income and degree of hearing loss), children enrolled at school were less likely to report difficulty making friends (aOR: 0.2; 95% CI: 0.1–0.6), while children were more likely to experience difficulty making friends if they had a speech impairment (aOR: 6.3; 95% CI: 2.3–17.4) or an illiterate caregiver (aOR: 3.1; 95% CI: 1.1–8.7; Table 3). Children having difficulty communicating needs was reported by 35.6% (107/301) of the caregivers. After eliminating non-significant variables (age, sex, illiterate caregiver, income and degree of hearing loss), having difficulty making friends was negatively associated with school enrolment (aOR: 0.2; 95% CI: 0.1–0.6) and living in Ntcheu (aOR: 0.4; 95% CI: 0.2–0.7), but more common among children with a speech impairment (aOR: 4.4; 95% CI: 2.1–9.2).

**Table 3:** Sociodemographic characteristics of children with hearing impairment whose caregiver reported their difficulty making friends or communicating needs, Malawi, 2016

Characteristic as recorded at baseline	Difficulty making friends (*n* = 30)					Difficulty communicating needs (*n* = 107)			
	No. followed-up (*n* = 299)^a^	No. (%)	cOR (95% CI)	aOR (95% CI)^b^		No. followed-up (*n* = 301)^c^	No. (%)	cOR (95%CI)	aOR (95% CI)^d^
**Age, years**									
0–4	63	6 (9.5)	Reference	NA		63	24 (38.1)	Reference	NA
5–9	95	7 (7.4)	0.8 (0.2–2.4)	NA		96	37 (38.5)	1.0 (0.5–2.0)	NA
10–14	88	9 (10.2)	1.1 (0.4–3.2)	NA		90	28 (31.1)	0.7 (0.4–1.5)	NA
15–18	53	8 (15.1)	1.7 (0.5–5.3)	NA		52	18 (34.6)	0.9 (0.4–1.9)	NA
**Sex**									
Female	132	11 (8.3)	0.7 (0.3–1.6)	NA		134	44 (32.8)	0.8 (0.5–1.3)	0.4 (0.2–0.7)
Male	167	19 (11.4)	Reference	NA		167	63 (37.7)	Reference	Reference
**District**									
Ntcheu	126	12 (9.5)	0.9 (0.4–2.0)	NA		128	30 (23.4)	0.4 (0.2–0.6)	0.4 (0.2–0.7)
Thyolo	173	18 (10.4)	Reference	NA		173	77 (44.5)	Reference	Reference
**School enrolment **									
Yes	180	13 (7.2)	0.2 (0.1–0.4)	0.2 (0.1–0.6)		181	59 (32.6)	0.2 (0.1–0.5)	0.2 (0.1–0.6)
No	30	10 (33.3)	Reference	Reference		30	21 (70.0)	Reference	Reference
Not of school age^e^	89	7 (7.9)	NA	NA		90	27 (30.0)	NA	NA
**Speech impairment**									
Yes	70	19 (27.1)	7.4 (3.2–17.2)	6.3 (2.3–17.4)		73	43 (58.9)	3.7 (2.1–6.5)	4.4 (2.1–9.2)
No	229	11 (4.8)	Reference	Reference		228	64 (28.1)	Reference	Reference
**Illiterate caregiver **									
Yes	107	13 (12.1)	1.3 (0.6–2.9)	3.1 (1.1–8.7)		107	37 (34.6)	0.9 (0.6–1.5)	NA
No	169	16 (9.5)	Reference	Reference		171	63 (36.8)	Reference	NA
Not recorded	23	1 (4.3)	NA	NA		23	7 (30.4)	NA	NA
**Income group, MWK**									
≤ 12 000	262	24 (9.2)	Reference	NA		265	89 (33.6)	Reference	NA
> 12 000	24	5 (20.8)	2.6 (0.9–7.7)	NA		23	13 (56.5)	1.5 (0.6–3.6)	NA
Not recorded	13	1 (7.7)	NA	NA		13	5 (38.5)	NA	NA
**Degree of hearing loss^f^**									
Mild	65	7 (10.8)	Reference	NA		64	18 (28.1)	Reference	NA
Moderate	59	5 (8.5)	0.8 (0.2–2.6)	NA		61	15 (24.6)	0.8 (0.4–1.6)	NA
Severe to profound	11	3 (27.3)	3.1 (0.6–15.0)	NA		11	7 (63.6)	3.0 (0.9–10.5)	NA
Bilateral otoacoustic emission failure	164	15 (9.1)	NA	NA		165	67 (40.6)	NA	NA

aOR: adjusted odds ratio; CI: confidence interval; cOR: crude odds ratio; MWK: Malawian Kwacha; NA: not applicable.

^a^ Data not recorded for eight children.

^b^ After eliminating non-significant variables (age, sex, district, income and degree of hearing loss).

^c^ Data not recorded for six children.

^d^ After eliminating non-significant variables (age, sex, illiterate caregiver, income and degree of hearing loss).

^e^ School age is defined as 6 years or older.

^f^ Categories of hearing loss are defined as: mild, 26–40 dB hearing level; moderate, 41–60 dB hearing level; severe to profound, 61 dB hearing level and greater.

Table 4 shows that 29.5% (72/244) of the school-aged children were not enrolled at school. After adjusting for non-significant variables (district, whether a speech impairment, income and degree of hearing loss), factors associated with lack of school enrolment were being in the two older age groups (10–14 years, aOR: 4.8; 95% CI: 1.9–12.1; 15–18 years, aOR: 28.6; 95% CI: 10.3–79.6), being female (aOR: 2.4; 95% CI: 1.2–4.8) or having an illiterate caregiver (aOR, 2.1; 95% CI: 1.0–4.1).

**Table 4:** Sociodemographic characteristics of school-aged children with hearing impairment who were not enrolled at school, Malawi, 2016

Characteristic as recorded at baseline	No. of school-aged children	No. (%) not enrolled at school	cOR (95% CI)	aOR (95% CI)^a^
Total	244	72 (29.5)	NA	NA
**Age, years**				
5–9	98	8 (8.2)	Reference	Reference
10–14	91	26 (28.6)	4.5 (1.9–10.9)	4.8 (1.9–12.1)
15–18	55	38 (69.1)	25.2 (7. 6–83.5)	28.6 (10.3–79.6)
**Sex**				
Female	107	39 (36.4)	1.8 (1.0–3.2)	2.4 (1.2–4.8)
Male	137	33 (24.1)	Reference	Reference
**District**				
Ntcheu	107	39 (36.4)	1.8 (1.0–3.2)	NA
Thyolo	137	33 (24.1)	Reference	NA
**Speech impairment**				
Yes	54	20 (37.0)	1.6 (0.8–2.2)	NA
No	190	52 (27.4)	Reference	NA
**Illiterate caregiver**				
Yes	85	30 (35.3)	1.6 (0.9–2.8)	2.1 (1.0–4.1)
No	139	36 (25.9)	Reference	Reference
Not recorded	20	6 (30.0)	NA	NA
**Income group, MWK**				
≤ 12 000	212	62 (29.2)	Reference	NA
> 12 000	22	8 (36.4)	1.4 (0.6–3.5)	NA
Not recorded	10	2 (20.0)	NA	NA
**Degree of hearing loss^b^**				
Mild	65	28 (43.1)	Reference	NA
Moderate	63	23 (36.5)	1.3 (0.6–2.7)	NA
Severe to profound	13	4 (30.8)	1.7 (0.6–6.2)	NA
Bilateral otoacoustic emission failure	103	17 (16.5)	NA	NA

aOR: adjusted odds ratio; CI: confidence interval; cOR: crude odds ratio; MWK: Malawian Kwacha; NA: not applicable.

^a^ After eliminating non-significant variables (district, whether a speech impairment, income and degree of hearing loss).

^b^ Categories of hearing loss are defined as: mild, 26–40 decibels (dB) hearing level; moderate, 41–60 dB hearing level; severe to profound, 61 dB hearing level and greater.

## Discussion

Less than half of the children identified with a hearing impairment at baseline were traced 3 years later, showing that mechanisms are needed to improve follow-up in the community. However, those lost to follow-up had similar baseline characteristics to those that were included at follow-up, reducing the potential for selection bias. Possible strategies to improve follow-up could include improving parental involvement and working together with established community structures, such as traditional leaders.^21^

Another challenge highlighted was the relatively low referral uptake, particularly for girls and younger children. Our results showed that referral uptake was higher among children living in Thyolo than in Ntcheu. Both districts are rural and poor, but Thyolo is closer to Blantyre, meaning children from Thyolo may have better access to health professionals and services than children from Ntcheu.

Another study in Malawi also showed that uptake of referrals was low among children with a hearing impairment, and reported that barriers include geographical accessibility, availability of services, affordability of transport and indirect costs, and acceptability (dependent upon knowledge and information about referral).^8^ To increase the availability of services in Malawi, the ear, nose and throat lead at the College of Medicine and colleagues developed and initiated relevant services and the training of clinical officers who are now serving in different districts of the country, including Ntcheu and Thyolo.^22^ Other potential interventions that could improve uptake of referrals include increased awareness of ear and hearing disorders, and the provision of transport and outreach services.^23^ Our findings suggest that illiteracy of the caregiver is an important predictor of lack of referral uptake and low participation outcomes in children with hearing impairment. For interventions in children with hearing loss to be effective, they should therefore be appropriate, timely and family-centred, and undertaken through an interdisciplinary approach (e.g. involving both traditional leaders and community health workers).^24^

As expected, many children with hearing loss had speech impairments and difficulties communicating their needs. Communication defines us and underlies our ability to function in the world. The ability to communicate effectively is essential for living independently, pursuing personal goals and interests, performing social roles and functions, maintaining personal and familial relationships, making decisions, and exercising control over quality of life and care.^25^ Our results show that children with communication difficulties were less likely to be enrolled at school and more likely to experience difficulties making friends. One way of improving speech or vocalization in children with hearing loss is the provision of speech therapy services, of which there is a shortage in sub-Saharan Africa; training programmes are needed to fill this gap.^6^

Our findings that one third of the school-aged participants were not enrolled at school are consistent with previous published studies showing that children with disabilities are less likely to attend and progress through school.^18^ However, children with hearing loss also have a right to education and should be encouraged to enrol in schools as part of the inclusive education strategy. Teachers in schools should be made aware of the needs of children with hearing loss, and the impact this disability has on a child’s ability to make friends and communicate needs. However, information is lacking as to what works best to improve educational outcomes among children with disabilities.^26^ These challenges are set against wider concerns about access to schooling in Africa. In Malawi, about 1.5 million out of 3.7 million (about 40%) of the children do not go beyond primary school education.^20^ This general pattern may explain our findings that older children with a hearing impairment were particularly unlikely to be enrolled at school.

Our study has limitations. We did not investigate school enrolment or participation outcomes in a comparison group of children without hearing loss. Furthermore, outcomes such as educational inclusion and difficulties making friends were recorded subjectively, and information from school records and exam results were not included. We did not develop mechanisms to make a link between the children seen at the screening camp and those seen by the ear, nose and throat specialist at the hospital, or obtain ethical approval for making this connection. This link would have been difficult to make without including additional mechanisms, as children often have multiple names and cannot be traced by name alone. Finally, the follow-up questionnaire was administered by a community health worker, raising the possibility of a positive-response bias for service satisfaction even though the health worker was not connected to the ear, nose and throat services.

Our study also has strengths, including the 3-year follow-up, the large cohort identified by our key informants and that children were recruited from the community rather than the clinic, improving the generalizability of the results. We used the International Classification of Functioning, Disability and Health for Children and Youth framework to assess outcomes, therefore looking holistically beyond functional status alone. With poor outcomes, in terms of referral uptake, social inclusion and well-being, for children with a hearing impairment in Malawi, more widespread and holistic services are required.
